# Childhood Trauma and Minimization/Denial in People with and without a Severe Mental Disorder

**DOI:** 10.3389/fpsyg.2017.01276

**Published:** 2017-08-24

**Authors:** Chelsea Church, Ole A. Andreassen, Steinar Lorentzen, Ingrid Melle, Monica Aas

**Affiliations:** ^1^School of Psychology, University of Glasgow Glasgow, United Kingdom; ^2^Division of Mental Health and Addiction, NORMENT K.G. Jebsen Centre for Psychosis Research, Oslo University Hospital and Institute of Clinical Medicine, University of Oslo Oslo, Norway; ^3^Institute of Clinical Medicine, University of Oslo Oslo, Norway

**Keywords:** childhood trauma, psychopathology, minimization, schizophrenia, bipolar disorder

## Abstract

**Background:** Childhood trauma has garnered extensive research concerning its role in the psychopathology of mental disorders, including psychosis. The Childhood Trauma Questionnaire (CTQ) utilizes a minimization/denial (MD) scale to denote potential under-reporters of trauma, yet MD scores are infrequently reported and validations of the scale are lacking in the literature. Study aim: Elucidate differences in MD between patients with severe mental disorders to healthy individuals, and secondly, investigate if MD influences reports of childhood trauma between the groups.

**Methods:** We included 621 patients with a DSM-schizophrenia spectrum, bipolar spectrum diagnosis, or major depression disorder with psychotic features and 299 healthy controls as part of the NORMENT study in Oslo, Norway. History of childhood trauma was obtained using the CTQ. Clinical diagnoses were assessed using the Structured Clinical Interview for DSM Disorders.

**Results:** A significantly greater proportion of healthy controls (42.8%) had a positive MD score compared to patients (26.7%). When controlling for MD, the patient group still exhibited elevated reports of childhood trauma compared to controls (Cohen’s *d* = 1.27), concordant with reports of childhood trauma being more frequently reported in a population of severe mental disorders.

**Conclusion:** Elevated MD in the healthy control group could suggest an enhanced self-serving bias, potentially attenuated in the psychiatric group. Clinicians and researchers would benefit from including the MD component of CTQ when assessing retrospective information on childhood trauma to rule out potential effect of MD.

## Introduction

A history of childhood trauma is reported at a much greater rate in severe mental disorders compared to healthy individuals (Etain et al., 2008, 2013; Fisher et al., 2010; Mondelli et al., 2010), linked to onset of psychotic experiences (McGrath et al., 2017) and more severe clinical features (Yung et al., 2015). The rate of child maltreatment in the general population has been estimated to 11% for sexual abuse and 24% for physical abuse (United Kingdom, May-Chahal and Cawson, 2005). This compares with numbers closer to 50% in patients with a psychotic illness comprised of both sexual and physical abuse (Read et al., 2005). Most studies, including the above, have used retrospective interviews, such as for example the Childhood Trauma Questionnaire (CTQ) to assess trauma experiences, asking adults about experiences in childhood. Ideally, a history of childhood trauma would be assessed using actual cohort records of childhood neglect and abuse in longitudinal studies. The study by Widom et al. (2005), compared cohort records of childhood neglect and abuse in longitudinal studies vs. self-reported measures of childhood neglect and abuse in retrospective studies showing good validity of the self-reported measures (Widom et al., 2005). Since, it is often not feasible to obtain actual reports of childhood abuse and neglect, the field needs a tool to measure self-report bias when assessing childhood trauma, especially for people with severe mental illness. Not much is known about potential confounders, such as differences in minimization and denial in retrospective reports of childhood trauma. In light of the retrospective nature of the CTQ, a response bias could weaken the validity of the measure. The minimization/denial (MD) scale is designed to detect a response bias that minimizes the extent of childhood trauma experienced. Minimization and denial measured by the MD scale consists of strongly agreeing with the following: “There was nothing I wanted to change in my family”; “I had the perfect childhood”; and “I had the best family in the world” (Bernstein and Fink, 1998). Although minimization has received some recent attention (MacDonald et al., 2015, 2016), research is still sparse regarding the validity of this measure; if differences in minimization are present across populations, or if the amount of “true” abuse/neglect reported depend on level of MD.

Retrospective reports of childhood trauma have been criticized for a tendency to under rather than over report childhood trauma experiences compared to other methods of assessment (health worker notes, sibling interviews, and so on) (Fisher et al., 2011). The MD scale could be used to quantify the potential effect of minimization on childhood trauma data collected retrospectively. The MD scale correlate with the Balanced Inventory of Desirable Responses (BIDRs) (a measure of social desirability) supporting the MD in detecting a social desirability bias (Bernstein and Fink, 1998). Social desirability is a cognitive process of editing relevant information in a socially desirable fashion (Holtgraves, 2004). Social desirability has, in addition to correlating with the MD scale, been linked to higher scores on the trait of self-deception (Holtgraves, 2004). Although the MD scale has been suggested to correlate with editing relevant information in a socially desirable fashion, the scale is rarely reported in the literature in studies of childhood trauma, and validation of the scale is sparse.

*The main aim* of the study is to investigate potential differences in MD in a large sample of schizophrenia spectrum disorders (SZ), patients with a bipolar spectrum disorder (BD), patients with major depressive disorder with at least one psychotic episode (*n* = 621) and healthy individuals (*n* = 299). All patients are part of the broader psychosis continuum disorders (Tesli et al., 2014). Study hypothesis: firstly (based on the study by MacDonald et al., 2015, 2016) we expect differences in MD scores between the patients group and the healthy control group. Based on similar trauma scores within the patient group (Etain et al., 2013; Aas et al., 2017) no differences in MD are expected within the patient group. Secondly, we hypothesized that patients with severe mental disorders will report more childhood trauma experiences than the healthy control group also after correcting for potential differences in MD style.

## Materials and Methods

### Participants

The participants were consecutively recruited from psychiatric units (outpatient and inpatient) in four major hospitals in Oslo as part of the larger NORMENT, Thematically Organized Psychosis (TOP) Research Study. The current study consists of patients recruited any time between 2007 and 2015, and controls recruited any time between 2011 and 2015. A total of 621 participants [with schizophrenia spectrum (*n* = 368), bipolar spectrum diagnoses (*n* = 253) or major depressive disorder with psychotic features (*n* = 24) and healthy individuals (*n* = 299)] were recruited. Within the schizophrenia spectrum group, the majority had a schizophrenia diagnosis [schizophrenia (*n* = 195), schizophreniform disorder (*n* = 27), schizoaffective disorder (*n* = 51), and psychoses not otherwise specified (NOS) (*n* = 95)]. Within the bipolar spectrum group the majority had a bipolar 1 diagnosis [bipolar 1 (*n* = 159), bipolar II (*n* = 54), and bipolar disorder NOS (*n* = 16)]. In addition, 24 patients had a diagnosis of major depressive disorder with psychotic features. A history of psychosis in affective patients was based on information retrieved from the SCID interview. The majority of the patients (70%; *n* = 437) were taking antipsychotic medication at the time of the assessment. In addition, 31% (*n* = 194) also used antidepressant medication. Furthermore, 27% (*n* = 169) of the patients were taking mood stabilizers at the time of the assessment. The mean age of the patients was 30.4 ± 10.6 years and 327 (53%) of them were males. Patients with a bipolar disorder with or without psychotic features were included in this study. Among patients with a bipolar I diagnosis, more than two thirds (69%) had at least one psychotic episode, while one third of them (31%) had no psychotic episode. Healthy control group of 299 participants was recruited from the same geographical areas as the patients. The healthy control group was similarly matched in age (mean ± SD: 30.1 ± 7.7) and in gender composition (56% male) as compared to the patient group (age: mean ± SD: 30.4 ± 10.6; 53% males). Both patients and healthy controls were assessed by trained psychiatrists or clinical psychologists. Healthy controls were screened with an interview to capture symptoms of severe mental illness [Primary Care Evaluation of Mental disorders (PRIME-MD); Spitzer et al., 1994]. To help counteract the effects of socio-economic differences between different parts of the city, controls were randomly recruited from the same city areas as patients. The healthy controls were randomly selected from statistical records^1^ from the same catchment area as the patients in the Oslo region. The exclusion criteria for all groups were an unstable or uncontrolled medical condition that interferes with brain function, and an age outside the range of 18–65 years. The Regional Committee for Medical Research Ethics and the Norwegian Data Inspectorate approved the study. All participants gave their written informed consent.

### Clinical Assessment

Trained psychiatrists, physicians and clinical psychologists carried out the clinical assessment, and a diagnosis was based on the research version of the Structured Clinical Interview for DSM-IV Axis I disorders (SCID-I). All patients were assessed on the modules A, B, C, D, and E. In addition, all raters finished a training course in SCID assessment based on the training program at the UCLA (Ventura et al., 1998). The diagnostic reliability was found to be satisfactory (Ringen et al., 2008), with an overall agreement for DSM-IV diagnostic categories of 82% and an overall κ of 0.77 (95% CI: 0.60–0.94).

### Childhood Trauma Questionnaire (CTQ)

Traumatic events in childhood were rated using a Norwegian version of the CTQ short version (Bernstein et al., 2003; Aas et al., 2014). This self-report questionnaire with 28 items (Bernstein et al., 2003) yields scores on five subscales of trauma on a Likert scale format, ranging from 1 to 5, ranging from never true, to very often true. The following five subscales were captured: emotional abuse (EA), physical abuse (PA), sexual abuse (SA), physical neglect (PN) and emotional neglect (EN), as well as a trauma total score as described in Bernstein et al. (1994, 2003). The CTQ also includes MD scale to detect underreporting of childhood trauma on the CTQ. Three reverse scored statements are rated on a Likert scale, with high minimization present if the participant would not change anything about their family, their family was the best in the world and they had the ‘perfect childhood.’ Selecting ‘very likely’ for any of these statements award one point, allowing a score of 0–3. Bernstein and Fink (1998) stated that any score above 0 indicated minimization. Any scores from 1 to 3 on the CTQ’s MD Scale suggests the possible underreporting of maltreatment (false negatives) (Bernstein and Fink, 1998). “No,” “low,” “intermediate,” and “high” minimization and denial corresponds to a MD score of 0–3. A MD score of “yes” correspond to at least one item that measures MD is scored as a 5 (“very often true”). “No” MD corresponds to no item score of 5 (“very often true”) on the three items covering MD on the CTQ. The reliability of the MD scale has previously been published in a large multicenter study (MacDonald et al., 2015, 2016), with reliability score of 0.77. In our sample a moderate to good internal consistency of the MD items were observed with a Cronbach’s alpha coefficient of 0.75. The validity of the MD scale has been estimated based on a high correlation with The BIDRs (Bernstein and Fink, 1998).

### Statistical Analyses

Data were analyzed using the Predictive Analytic Software (PASW), Version 21 (formerly SPSS Statistics). The sample was divided into a minimization vs. no minimization group, with minimization operationally defined as a score of 1 or greater on the MD scale. Grading severity of MD (scores rating from 0 to 3) was also included. Differences in categorical variables (gender, diagnosis, and group status) between ‘minimizers’ and ‘non-minimizers’ were tested using the chi-square test. As the childhood trauma data were skewed, Mann–Whitney *U*-test was performed to assess CTQ scores in minimizers and non-minimizers with CTQ measured as a continuous variable. For the follow-up analysis, childhood trauma was dichotomized into two groups based on at least one subdomain of childhood trauma reaching levels of moderate to severe reports following the definition by Bernstein et al. (1994, 2003) (see Supplementary Table S1).

Effect sizes were computed using Cohen’s *d* (Cohen, 1977). For the effects sizes we compared trauma scores in the patients compared to the control group (Cohen’s *d* = *M*_1_–*M*_2_/s_pooled_, where s_pooled_ = √[($s_{1}^{2}$ + $s_{2}^{2}$)/2]). According to Rosenthal and Rosnow (1984), effect sizes were considered small for values between 0.20 and 0.50, moderate for values between 0.50 and 0.80, and large for values greater than 0.80. Logistic regression was performed to investigate differences in reports of childhood trauma (reaching above cutoff score for moderate to severe trauma on at least one subdomain) and group status (patients/controls), correcting for MD. Childhood trauma (yes/no) was entered as the dependent variable; MD (yes/no) and group status (patients/controls) as independent variables with a pre-set significance level of 0.05.

## Results

### Demographics of the Sample According to MD Score

Selected demographics of the minimization (MD) and non-minimization (no MD) groups are presented in **Table 1**. Of the 920 study participants, 32% (*N* = 294) demonstrated a MD score and 68% (*N* = 626) did not. Neither gender (*P* = 0.98), patients group (*P* = 0.70) nor age (*P* = 0.87) had a significant association with MD score (see **Table 1**).

**Table 1:** Demographics of the sample divided into presence of minimization.

	No minimization (*N* = 626)		Minimization (*N* = 294)		Statistics	
	*N*	%	*N*	%		
Gender (M/F)	337/289	53.8/46.2	158/136	53.7/46.3	X^2^= 0.001, df = 1	*P* = 0.98
Diagnosis					X^2^= 0.73, df = 2	*P* = 0.70
Schizophrenia	265	58.2	103	62.0		
Bipolar disorder	172	37.8	57.0	34.3		
Major depressive disorder with psychotic features	18	4.0	6	3.6		
Group					X^2^= 23.99, df = 1	*P* < 0.001
Patients	455	73.3	166	26.7		
Controls	171	57.2	128	42.8		
Medication						
Antipsychotics *n* (%) Antidepressant *n* (%) Mood stabilizer *n* (%)	314 141 117	50.2 22.5 18.7	123 53 52	41.8 18.0 17.7	X^2^= 1.51, df = 1 X^2^= 0.05, df = 1 X^2^= 1.93, df = 1	*P* = 0.21 *P* = 0.82 *P* = 0.16
	**Mean**	***SD***	**Mean**	***SD***		
Age	30.2	9.7	30.4	9.8	*Z* = -0.17	*P* = 0.87

*Skewed data were measures using the Mann–Whitney test.*

### Childhood Trauma and MD Score

**FIGURE 1:**
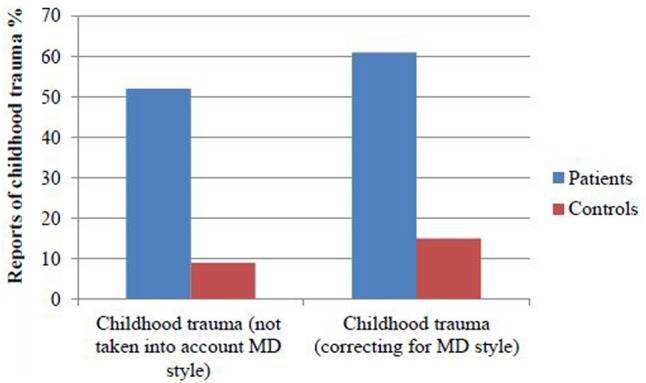
Fifty one % of the patients reported at least one subdomain of childhood trauma, compared to 9% of the controls. When taking into account minimization 61% of the patients reported at least one subdomain of childhood trauma compared to 15% of the healthy controls.

42.8% of the controls had a MD score of ≥1 compared to 26.7% of the patients (X^2^= 23.99, *P* < 0.001). 51% of the patients reported at least one subdomain of childhood trauma (≥moderate to severe score on CTQ), compared to 9% of the controls (X^2^= 148.0, df = 1, *P* < 0.001, Cohen’s *d* = 1.18). Excluding participants with MD scores ≥ 1, 61% of patients reported at least one subdomain of childhood trauma (≥ moderate to severe score on CTQ) compared to 15% of the healthy controls (X^2^= 99.3, df = 1, *P* < 0.001, Cohen’s *d* = 1.27, see **Figure 1**). In the patient group, a MD score of ≥1 was associated with lower CTQ score, compared to the patients with MD < 1 (Mann–Whitney test: *Z* = -10.66, *P* < 0.001). Also in the controls having a MD score of ≥1 was associated with lower CTQ score, compared to controls with MD < 1 (Mann–Whitney test: *Z* = -9.29, *P* < 0.001). Dividing into subdomains of childhood trauma, minimizers (MD score ≥ 1) presented significantly lower CTQ scores across all subtypes of trauma compared to patients and controls with MD < 1 (see Supplementary Figures S1, S2, and Table S2), with the most significant findings for emotional neglect.

A significant difference in childhood trauma (yes/no [defined as at least one subdomain of ≥moderate to severe score on CTQ]) was observed between patients and controls also after controlling for MD [Nagelkerke *r*^2^ = 0.33; exp (B) = 0.102, *P* < 0.001, see **Table 2**].

**Table 2:** Patients reported more childhood trauma than healthy controls also after correcting for MD.

Childhood trauma	*B*	*SE*	Wald	df	Significance	Exp (B)	95% C.I. for Exp (B)	
							Lower	Upper
MD (yes/no)	2.39	0.75	10.27	1	0.001	10.89	2.53	46.93
Patient/control status	3.09	0.74	17.61	1	<0.001	21.98	5.19	93.08
Constant	–4.11	0.71	33.25	1	<0.000	0.02		

*^∗^Logistic regression; Childhood trauma (yes/no) is defined as least one subdomain of trauma above cutoff score for moderate to severe trauma; MD style (yes/no) is defined as the following: “MD style yes” = minimizers,” score of ≥1 on the MD scale, and “MD style no” = “no minimization” score of 0 on the MD scale. Nagelkerke (*r*^*2*^) = 0.33.*

**FIGURE 2:**
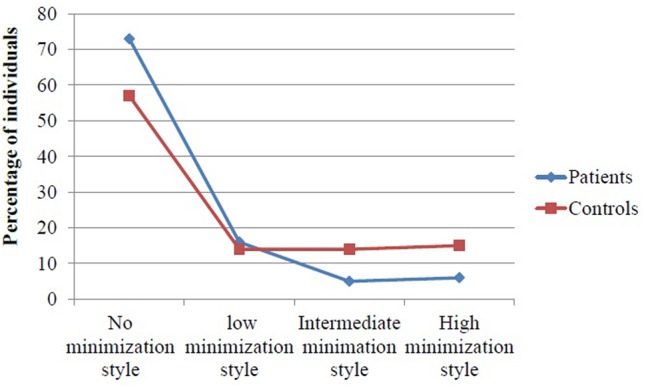
Controls report increased minimization style measured by the minimization/denial (MD) scale compared to patients.

Dividing into no, low, intermediate, and high minimization and denial (MD score of 0–3 respectively), controls had more frequently a score of intermediate or high MD compared to patients (X^2^= 48.7, df = 1, *P* < 0.00.1 see **Figure 2**).

## Discussion

To our knowledge the current study is one of the first studies to investigate minimization and denial by the MD scale and retrospective reports of childhood trauma amongst healthy individuals and in individuals with a severe mental disorder (SZ, BD, or major depression disorders with at least one psychotic episode). In our study the MD score was elevated in healthy individuals compared to the patient group. A significant negative association was observed between MD and CTQ scores. When we removed participants with MD scores ≥ 1, patients still reported significantly more childhood trauma experiences than the healthy control group (Cohen’s *d* = 1.27). No difference in MD was observed within the patient sample (schizophrenia spectrum disorder, patients with a bipolar disorder, or patients with major depressive disorder with at least one psychotic episode). When we examined the impact of MD on CTQ subscale scores, we found the largest effect for patients and controls on the CTQ emotional neglect subscale. Similar findings have been reported in the study by MacDonald et al. (2016). Thus, endorsement of MD seems to be specifically sensitive to emotional neglect. It seems that those with emotional neglect would be less likely to claim that they had a perfect family. As discussed in the paper by MacDonald et al. (2016) reasons for this may include content overlap (for example four of the five items comprising the emotional neglect subscale contain the word “family” as compared to two of the three items comprising the MD score).

Our findings support evidence of a higher prevalence of childhood trauma in patients with severe mental disorders than in the normal population, as consistently reported in the literature (Etain et al., 2008, 2013; Fisher et al., 2010; Mondelli et al., 2010). Similar to the large multicentre study by MacDonald et al. (2016) comprised of healthy individuals and various psychiatric patients, we found that patients had lower minimization than the healthy individuals. This could be due to a larger proportion of individuals without a mental illness to recall life events with a ‘rosy view’ (Mitchell et al., 1997), a positivity bias recall not demonstrated for example by depressed individuals (Ben-Zeev and Young, 2010). This positivity bias could be a reason for elevated MD amongst healthy individuals in our study, with the reality of traumatic childhood experiences selectively underreported to maintain what Heider coined as the ‘individuals positive outlook’ (Heider, 1958). This positivity illusion has been repeatedly characterized as a typical cognitive mechanism among healthy individuals in Western cultures (Taylor and Brown, 1994; Greenwald et al., 2002), serving a purpose to preserve mental health. The human desire for esteem and need to view oneself positively form an important function in our psychological self-preservation (Baumeister and Leary, 1995). This may also involve seeing significant others, such as parents, in a more positive light. A meta-analysis of 266 studies support a significantly smaller self-serving attributional bias in psychopathological samples (Cohen’s *d* effect size = 0.48) compared to individuals with no psychopathology (Cohen’s *d* = 1.28) (Mezulis et al., 2004). Based on the above, we suggest more studies are needed to investigate if differences in self-serving attribution style influence responses to retrospective questionnaires and interviews across different population groups.

### Study Limitations

Childhood trauma was collected using the CTQ, a retrospective measure of childhood trauma experiences with the inherited weakness of its retrospective design. However, retrospective information on childhood trauma is a frequently used measure with high reliability and validity in a psychotic population (Fisher et al., 2011). Reports of childhood trauma has been found stable over time (test–retest reliability) in addition to a large overlap of reports of childhood trauma across different sources [i.e., clinical case notes, questionnaires (convergent validity) (Fisher et al., 2011)]. The validity of the MD measure needs further investigations. We did not have any data on social desirability or attribution style, therefore we can only speculate that differences in minimization between our groups were based on differences in social desirability and attribution style. A further limitation is the failure to assess for the presence of Axis II personality disorders. The high likelihood of unacknowledged Axis II disorders may underestimate the effect of MD. It is well-documented that individuals with a personality disorder, particularly Cluster B (borderline, histrionic, narcissistic, or antisocial) more often report a history of abuse (Molnar et al., 2001). It is likely personality disorder diagnoses were present in the psychiatric population, due to their high comorbidity with Axis I diagnoses (Links and Eynan, 2013). The presence of personality disorder diagnoses within the patients’ sample may have impacted both CTQ scores and levels of minimization and denial of past-trauma.

## Conclusion

Higher MD scores were notably observed in the healthy control group which could be based on enhanced self-serving bias, potentially attenuated in the psychiatric group. Clinicians and researchers would benefit from including the MD component of CTQ when assessing retrospective information of childhood trauma to rule out potential effects of MD.

## Author Contributions

MA, CC, OA, SL, and IM contributed to the study design and writing up process.

## Conflict of Interest Statement

The authors declare that the research was conducted in the absence of any commercial or financial relationships that could be construed as a potential conflict of interest.

## Footnotes

1 www.ssb.no
