# Supplementary material for: Childhood Trauma and Minimization/Denial in People with and without a Severe Mental Disorder
Source: Front Psychol. 2017 Aug 24;8:1276. doi: 10.3389/fpsyg.2017.01276 (PMC5573805; doi:10.3389/fpsyg.2017.01276)
Supplement: Supplementary file 1 [file Table_1.DOC]

CTQ moderate to severe cutoff score for abuse

| CTQ, Childhood abuse subtypes | Moderate to severe cutoff |
| --- | --- |
| Physical abuse | ≥10 |
| Sexual abuse | ≥8 |
| Emotional abuse  Physical neglect  Emotional neglect | ≥13  ≥10  ≥15 |

For estimates of frequencies of childhood abuse we used the moderate to severe predefined cutoff suggested by Bernstein (Bernstein and Fink, 1998).
