# Supplementary material for: Childhood Trauma and Minimization/Denial in People with and without a Severe Mental Disorder
Source: Front Psychol. 2017 Aug 24;8:1276. doi: 10.3389/fpsyg.2017.01276 (PMC5573805; doi:10.3389/fpsyg.2017.01276)
Supplement: Supplementary file 2 [file Table_2.doc]

Patients and controls with MD have lower CTQ scores

|  | | Total CTQ score | Physical abuse | Sexual abuse | Emotional abuse | Emotional neglect | Physical neglect |  |
| --- | --- | --- | --- | --- | --- | --- | --- | --- |
| Patients | Mann-Whitney U | 13402.00 | 29921.50 | 28701.50 | 17710.00 | 13659.00 | 23142.50 |  |
|  | Wilcoxon W | 24878.00 | 43451.50 | 41581.50 | 30751.00 | 26700.00 | 36508.50 |  |
|  | Z | -10.66 | -4.11 | -4.53 | -9.53 | -11.69 | -7.07 |  |
|  | Asymp. Sig. (2-tailed) | *P*<0.001 | *P*<0.001 | *P*<0.001 | *P*<0.001 | *P*<0.001 | *P*<0.001 |  |
| Controls | Mann-Whitney U | 3748.00 | 9767.00 | 10265.50 | 6290.50 | 4025.50 | 9071.50 |  |
|  | Wilcoxon W | 11498.00 | 17895.00 | 18393.50 | 14546.50 | 12026.50 | 17199.50 |  |
|  | Z | -9.291 | -2.79 | -2.22 | -6.76 | -9.39 | -2.88 |  |
|  | Asymp. Sig. (2-tailed) | *P*<0.001 | *P*=0.01 | *P*=0.03 | *P*<0.001 | *P*<0.001 | *P*=0.004 |  |
| MD score 0=No minimization; 1=≥1 MD score. | | | | | | | | |
