# Supplementary figures and images for: Childhood Trauma and Minimization/Denial in People with and without a Severe Mental Disorder
Source: Front Psychol. 2017 Aug 24;8:1276. doi: 10.3389/fpsyg.2017.01276 (PMC5573805; doi:10.3389/fpsyg.2017.01276)

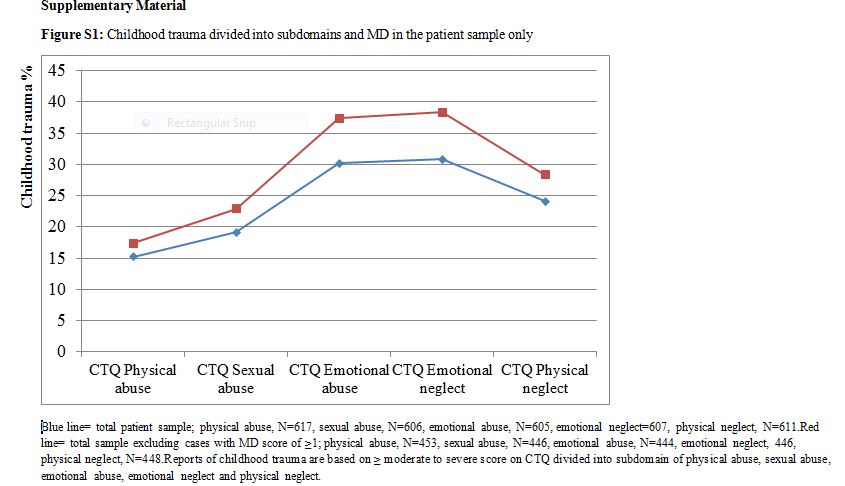

Supplement: Supplementary file 3 [file Image_1.JPEG]

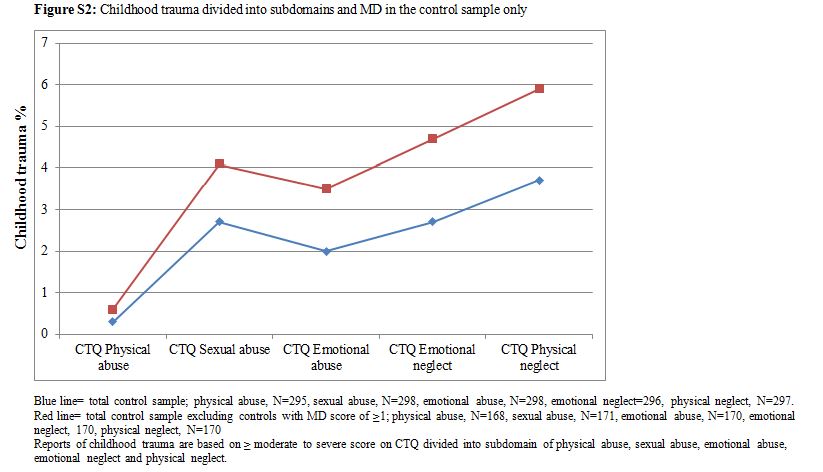

Supplement: Supplementary file 4 [file Image_2.JPEG]
